# Bariatric Surgery and Metabolic Status

**DOI:** 10.3390/medicina60091532

**Published:** 2024-09-20

**Authors:** Anna Różańska-Walędziak, Krzysztof Wyszomirski, Małgorzata Kaszuba, Anna Mierzejewska, Ewa Skopińska, Maciej Walędziak

**Affiliations:** 1 Department of Human Physiology and Pathophysiology, Faculty of Medicine, Collegium Medicum, Cardinal Stefan Wyszynski University, 01-938 Warsaw, Poland; aniaroza@tlen.pl (A.R.-W.); k.wyszomirski@uksw.edu.pl (K.W.); a.mierzejewska@uksw.edu.pl (A.M.); e.skopinska@uksw.edu.pl (E.S.); 2 Military Institute of Medicine, National Research Institute, Szaserów 128 St., 04-141 Warsaw, Poland; kaszuba.malgorzata@o2.pl; 3 Department of General, Oncological, Metabolic and Thoracic Surgery, Military Institute of Medicine, National Research Institute, Szaserów 128 St., 04-141 Warsaw, Poland

**Keywords:** obesity, bariatric surgery, eating habits, biochemical parameters, metabolic parameters

## Abstract

*Background and Objectives*: Obesity is associated with numerous co-morbidities, including dyslipidemia, insulin resistance and diabetes mellitus. Bariatric surgery is the mainstay of treatment for obesity as the only method with confirmed long-term effects in weight reduction and the remission of comorbidities. Postoperative recommendations leading to changes in dietary habits and changes in digestion and absorption in the gastrointestinal tract after bariatric surgery may additionally influence the levels of laboratory parameters that reflect the metabolic and nutritional status. The purpose of the study was to analyze the possible influence of changes in dietary habits after bariatric surgery on those laboratory results that reflect the metabolic and nutritional status. *Materials and Methods:* This was a retrospective study of 88 patients with a history of bariatric surgery. Data were gathered from before the surgery and at 6 months after the surgery and included diet structure and selected laboratory parameters reflecting the metabolic and nutritional status, i.e., levels of fasting glucose, glycated hemoglobin, cholesterol, low- and high-density lipoproteins, triglycerides, alanine and aspartate aminotransferases, proteins, ferrum, ferritin, vitamin B12, folic acid, vitamin D and calcium, the red blood cell count and the hematocrit. *Results*: Postoperative festive glucose levels were reduced by 14% and were more significant in patients after Roux-en-Y gastric bypass. There was an increase of 22% in concentrations of high-density lipoproteins. Triglyceride concentrations were reduced by 32%. Aminotransferase levels decreased by 43% for alanine aminotransferase and by 14% for aspartate aminotransferase. Among the changes in dietary habits, post-bariatric patients had a reduced consumption of red meat and an increased consumption of fish, milk and dairy products and wholegrain products. Vitamin D and ferrum levels were higher after the surgery, whereas vitamin B12 and folic acid levels remained unchanged. *Conclusions*: Improved dietary habits of patients after bariatric surgery may lead to changes in laboratory parameters that reflect the ameliorated metabolic and nutritional status of patients after bariatric surgery.

## 1. Introduction

The prevalence of obesity worldwide has been increasing alarmingly for the last few decades. According to the World Health Organization (WHO), presently more than 1.9 billion people worldwide are overweight, and 650 million suffer from obesity [1,2]. Obesity is associated with a large number of comorbidities, among which are dyslipidemia, insulin resistance, diabetes mellitus type 2, cardiovascular disease, hypertension and obstructive sleep apnea [3]. Obesity is associated with higher risk of both morbidity and mortality [4]. Bariatric surgery is the mainstay of treatment for obesity as it is the only method with confirmed long-term effects as measured by weight reduction and the remission of comorbidities [5,6]. Bariatric surgery should be accompanied by lifestyle changes, including a healthy diet and regular physical activity. The multidisciplinary team should include a psychologist, dietitian and physiotherapist [6,7].

Dietary regimens for bariatric patients start before the operation, when patients are encouraged to change dietary habits to better adjust to the dietary changes necessary after the surgery [8,9]. A reduction in body weight additionally reduces the risk of perioperative complications [10,11,12,13,14]. A preoperative dietary regimen should be prepared in cooperation with a bariatric dietitian, as malnutrition is common in patients with obesity [13,15]. Early re-alimentation, recommended in enhanced recovery after bariatric surgery (ERABS) protocols, reduces the risk of perioperative complications and the length of hospital stay [1]. After the surgery, the patient has to go through several stages of a dietary regimen from fluids to solids of different consistencies, and achieving optimum results requires consistency and constant compliance with the dietitian’s recommendations. Bariatric procedures lead to changes in digestion and absorption in the gastrointestinal tract, which may result in protein, micronutrient and vitamin deficiencies. Regular supplementation is of the utmost importance and should be provided for all patients to avoid the consequences of deficiencies [16,17,18,19].

Dietary recommendations and changes in digestion and absorption in the gastrointestinal tract after bariatric surgery may influence the levels of laboratory parameters that reflect the metabolic and nutritional status.

### Purpose of the Study

The purpose of the study was to analyze the influence of bariatric surgery and potentially associated changes in dietary habits on selected laboratory parameters that represent the metabolic status of the organism.

## 2. Materials and Methods

This was a retrospective study of patients who had a history of bariatric surgery in a tertiary referral bariatric center in 2-year period before the study. The protocol of the study was accepted by the Bioethics Committee of the Military Institute of Warsaw (nr 35/2023 11 April 2023). The inclusion criteria were the following: a history of bariatric surgery, age of 18 years old or more and available data on laboratory parameters and dietary habits.

Data were gathered from the patient’s medical documentation and included baseline characteristics and demographic data, body weight, Body Mass Index (BMI), laboratory parameters measured at two time-points (before the surgery and 6 months after the surgery) and dietary questionnaires routinely filled in by all the patients of the bariatric center before the operation and during a control visit 6 months after the surgery. Questions in the dietary questionnaires consisted of modules about the frequency of consumption of selected groups of alimentary products.

The analyzed laboratory parameters included glucose levels, measured by glycated hemoglobin (HbA1c; normal range: 29–41 mmol/mol) and fasting glucose (FGC; concentration (3.9–5.5 mmol/L), and parameters reflecting lipid metabolism, namely low-density lipoproteins (LDLs; <116 mg/dL), high-density lipoproteins (HDLs; >40 mg/dL), triglycerides (TGIs; <150 mg/dL) and total cholesterol (TC; <190 mg/dL). Additionally, the analyzed parameters included concentrations of alanine aminotransferase (ALT; 0–50 U/L), aspartate aminotransferase (AST; 0–50 U/L), total protein (TP; 6.4–8.3 g/dL) and hemoglobin (HGB; 13.5–17.0 g/dL), the red blood cell count (RBC; 4.36—5.78 T/L) and the hematocrit (HCT; 40–51%). The micronutrients and vitamins that were analyzed in the study were total calcium (TCA; 8.6–10.2 mg/dL), total ferrum (TFE; 59–158 µg/dL), ferritin (FER; 30–400 ng/mL), folic acid (FA; 4.5–37.3 ng/mL), vitamin D3 (D3; 20–80 ng/mL) and vitamin B12 (B12; 191–663 ng/mL).

The analyzed groups of alimentary products included red meat, poultry, milk and dairy products, egg, fish and seafood, soya and soya products, wholegrain products and brassica vegetables.

### Statistical Analysis

Statistical analysis was performed using the R studio program by RStudio Team 2020. The Mann–Whitney U test and Student *t* tests were used for quantitative data comparison between two groups as required. The Spearman correlation coefficient was used to assess the linear correlation between two variables. The two-sided Fisher’s exact test and chi-square test were used for categorical and binary data comparison as required. A *p* value < 0.05 was considered significant.

## 3. Results

The study group included 88 patients with a history of LSG or LRYGB in the 2 years before the study, with a median age of 37.8 years. A total of 87.5% of participants had a history of LSG. Baseline characteristics of the study group are presented in Table 1.

The average BMI before the surgery was 45.4 kg/m^2^ (SD 7.5), and the average body weight before the surgery was 131 kg (SD 29.9). The median %TWL after 6 months was 27.5%. There were no lost-to-follow-up patients; all patients were seen at each time point. Selected laboratory parameters before and 6 months after the surgery are presented in Table 2.

### 3.1. Dietary Habit Changes

Changes in dietary habits were observed in the frequency of consumption of different groups of alimentary products. There was a 5-fold decrease in the rate of patients who consumed red meat from 3 to up to 6 times a week and a 3-fold increase in the rate of patients who consumed red meat only 1 to 3 times a month. There was a 2-fold increase in the rate of patients who indicated that they consumed milk and dairy products 2 to 3 times daily. There were also significant changes in the consumption of fish, with a 41% decrease after the surgery in the rate of patients who chose sea fish only 2 to 3 times a month, and a simultaneous increase of 63% in the rate of patients who chose sea fish 1 to 2 times a week. The increase was also present in the consumption of freshwater fish. Detailed results concerning the changes in dietary habits are presented below.

### 3.2. Red Meat

There was a significant reduction in frequency consumption of red meat after the surgery. Before the surgery, 55.7% of patients reported the consumption of red meat 3 to 6 times a week, whereas only 11.4% did so after the surgery (*p* < 0.05). Simultaneously, the rate of patients choosing red meat only 1 to 3 times a month increased from 10.1% to 35.2% (*p* < 0.05).

### 3.3. Milk and Dairy Products

There was a significant increase in the consumption of milk and dairy products. Before the surgery, 31.8% of the study group indicated the consumption of milk and dairy products 2 to 3 times daily; this proportion almost doubled after the surgery, with 60.2% consuming milk and dairy products 2 to 3 times daily (*p* < 0.05). There were no changes observed in the consumption of eggs before and after the surgery.

### 3.4. Fish

There was also an increase of consumption of both freshwater and sea fish after the surgery. Before the surgery, 52.3% of participants indicated the consumption of sea fish 2 to 3 times a month, while 20.5% consumed sea fish 1 to 2 times weekly, whereas after the surgery, the proportions were 30.7% and 50%, respectively, (*p* < 0.05). The rate of patients who chose freshwater fish as part of their diet 2 to 3 times a month was 30.7% before the surgery, and this increased to 52.3% after the surgery; likewise, the consumption of freshwater fish 1 to 2 times weekly increased from 6.8% to 25% (*p* < 0.05).

### 3.5. Wholegrain Products

Before the surgery, 44.3% of participants reported the consumption of wholegrain products 2 to 5 times daily, which decreased after the surgery to 14.8% (*p* < 0.05). Simultaneously, the proportion of patients who indicated the consumption of wholegrain products once a day increased from 35.2% before the surgery to 65.9% in the post-bariatric group (*p* < 0.05).

There were no significant changes found in the consumption of soya and soya products before and after the surgery.

### 3.6. Laboratory Parameters

There was a significant reduction in both the glycated hemoglobin concentration and the fasting glucose plasma concentration of 9% and 14%, respectively, in the whole study group. Median LDL concentrations were reduced by 9% 6 months after the surgery, and the median HDL increased by 22%. The most significant reduction of 32% was observed in triglyceride levels. Detailed results are presented below.

### 3.7. Glucose

There was a significant decrease in HbA1c plasma concentrations after the surgery, with a median concentration of 39 mmol/mol before and 33 mmol/mol after the surgery (*p* < 0.05). The median FGC was 5.6 mmol/L in the pre-bariatric group and 4.8 mmol/L in he post-bariatric group (*p* < 0.05). 

### 3.8. Aminotransferases and Total Protein

Median ALT levels decreased from 29 U/L before the surgery to 17 U/L after the surgery, and AST also decreased from 22 U/L to 19 U/L (*p* < 0.05). Median TP levels increased from 5.9 g/L before the surgery to 7 g/L after the surgery (*p* < 0.05).

### 3.9. Lipids

There was no significant change observed in the total cholesterol levels before and after the surgery; however, there were significant changes in other lipid concentrations. The median LDL concentration was 113 mg/dL in the pre-bariatric group and 104 mg/dL in the post-bariatric group (*p* < 0.05). Median HDL levels increased from 48 mg/dL before the surgery to 58 mg/dL after the surgery (*p* < 0.05). 

There was a significant reduction observed in triglyceride levels, from 152 mg/dL to 104 mg/dL (*p* < 0.05).

### 3.10. Vitamins and Micronutrients

There was an increase observed in TFE levels, from 95 mg/dL before the surgery to 110 mg/dL 6 months after the surgery (*p* < 0.05). The median RBC decreased from 4.9 T/L in the pre-bariatric group to 4.6 T/L in the post-bariatric group (*p* < 0.05). There was a reduction from 42% to 41% observed in the median HCT; however, it was not statistically significant. There were no significant changes in ferritin and hemoglobin levels.

The median vitamin D3 concentration increased from 23.4 ng/mL before the surgery to 32.4 ng/mL after the surgery (*p* < 0.05). There were no statistically significant changes in total calcium, folic acid or vitamin B12 when analyzed in the whole study group.

## 4. Discussion

In our study, we analyzed the changes in the laboratory parameters representing the glucose and lipid levels after bariatric surgery. The purpose of the study was to observe whether changes in the digestive tract resulting from the surgery and postoperative dietary habits could have an influence on the optimization of laboratory parameters that indicate the metabolic status of the organism. 

We observed the amelioration of markers indicating the status of both carbohydrate and lipid metabolism, with a significant reduction in both glycated hemoglobin levels and fasting glucose plasma concentrations of 9% and 14%, respectively. The lipid profile that was used in our study was the basic lipid profile as presented in the new Polish recommendations and included total cholesterol, LDL, HDL and triglycerides [20]. Median LDL concentrations were reduced by 9% 6 months after the surgery, and median HDL levels increased by 22%. The most significant reduction of 32% was observed in triglyceride levels. The positive changes in the metabolic status, as measured by the laboratory parameters, can be attributed mainly to the influence of bariatric surgery on digestion and absorption in the digestive tract, weight reduction and the remission of comorbidities, but these can also be associated with dietary habit changes after the surgery.

Lipid parameter changes after bariatric surgery have been the subject of many studies. Genua et al. analyzed changes in lipid laboratory parameters after bariatric surgery. They observed maximum concentrations of HDL 2 years after the surgery and a decrease in HDL levels 3 months after the surgery compared with the pre-surgical levels. In the study by Genua et al., the increase in HDL levels was more significant after sleeve gastrectomy (SG) than after Roux-en-Y gastric bypass (RYGB) [21]. In our study, significant changes in HDL levels were not observed in patients after RYGB; however, our results were limited by the low number of patients after RYGB in the study group.

In our study, we observed changes in the dietary habits of patients after bariatric surgery as measured by changes in the consumption of selected groups of alimentary products, with a significant reduction in the consumption of red meat and an increase in the consumption of milk and dairy products and sea and freshwater fish. Changes in dietary habits were also observed in other studies on the alimentation of post-bariatric patients. Heusschen et al. analyzed the dietary preferences of 107 patients after bariatric surgery: 87 after LRYGB and 20 after LSG [22]. The reversed structure of the study group compared with our study can be attributed to the proportion of bariatric procedures performed in the Netherlands, where the majority of bariatric procedures are LRYGB, whereas in Poland, it is LSG [23]. Heusschen et al. analyzed dietary changes at the same time-point, i.e., 6 months after the surgery, and found changes which they estimated as being both positive and negative. There was a reduction in the consumption of red meat, which is in accordance with the results of our study, as well as of sweets, high-calorie snacks and sodium chlorate, and an increase in the consumption of milk and dairy products. However, there was a decrease in the consumption of vegetables, wholegrain products, fiber and micronutrients and an increase in the consumption of carbohydrates [22].

Bariatric surgery, and possibly the associated dietary habit changes, can also influence liver function. In our study, we observed a reduction in aminotransferase levels, with a decrease of 14% in median AST and 43% in median ALT levels. Our results remain coherent with those presented in other studies. Toman et al. found a positive influence of bariatric surgery on the remission of non-alcoholic fatty liver disease, presently known as metabolic-dysfunction-associated steatotic liver disease (MASLD) [24]. Zadeh et al. analyzed a group of 151 patients after bariatric surgery and found a significant reduction in AST and ALT concentrations after bariatric surgery, which was more distinct after LRYGB than LSG [25]. Conversely, there are studies that report no significant changes in aminotransferase levels during long-term observation. In a study by Samani et al. in a group of 40 patients 5 years after bariatric surgery, there were no changes in aminotransferase levels compared with pre-surgical results [26].

The influence of bariatric surgery on postoperative weight loss is a result of several mechanisms. Firstly, the reduction in gastric volume allows only for reduced food intake and, therefore, is responsible for the reduction in caloric intake. Additionally, a small gastric volume leads to fast stomach drainage, causing accelerated bowel movement. The energy balance is negative, and body weight is reduced. The next mechanism is the reduction in the number of gastric cells producing ghrelin, the central hormone responsible for the sense of hunger. The decrease in ghrelin levels is achieved through resection of the stomach fundus. Finally, procedures involving gastro-iliac anastomoses reduce the surface absorption of nutrients, reducing the total absorption in the intestine.

Bariatric surgery, through interference with the processes of digestion and absorption, also influences the levels of different micronutrients and vitamins, which is additionally modified by presence or absence of adequate supplementation. In our study, there was a significant increase of 39% in the vitamin D3 concentration in the whole study group, with a distinct difference between the sexes, with a 63% increase in men and 35% in women. This might result from more common general supplementation with vitamin D3 in women due to osteoporosis prophylaxis [27,28,29]. Vitamin D3 deficiency has different prevalence in different populations; therefore, the results of available studies on the subject are not coherent.

Povaliaeva et al., in a study comparing vitamin D3 levels between 30 patients with a history of bariatric surgery with 30 healthy individuals, found the presence of significant vitamin D3 deficiencies even before surgery. The level of deficiency could be reduced through intensive supplementation; however, the level of vitamin D3 remained suboptimal 3 months and 6 months after the surgery [30]. Javanainen et al. compared a large group of patients with a history of bariatric surgery (253 after LRYGB and 142 after LSG) with a group of 199 patients with obesity who had only lifestyle intervention. Follow-up at time-points of 12 months and 24 months revealed that even though patients after bariatric surgery had vitamin D3 levels within the normal range, they were at a higher risk of cumulative fractures than the patients in the control group [31]. Rashnoo et al. found vitamin D3 levels in a group of 120 patients 12 months after LSG to be higher than before the surgery [32]. Conversely, in a group of 67 patients with a history of LRYGB or LSG, Vinolas et al. found that vitamin D3 deficiency was still present in 67% of the patients 12 months after bariatric surgery [8].

There were no statistically significant differences in vitamin B12 levels in our study. The results differ between the available studies. Other researchers presented vitamin B12 levels to be within the normal range at follow-up points of 12 months and 24 months in a group of patients after bariatric surgery [30]. Guo et al. analyzed vitamin B12 levels in a group of 199 patients 1 month after the surgery and found them to be elevated in 56% of patients [33].

Bariatric surgery is associated with a risk of different micronutrient and vitamin deficiencies, the most common of which is ferrum deficiency, with a prevalence of more than 20% in the population of post-bariatric patients [34]. In our study, there was a significant increase of 16% in median ferrum concentrations and a non-significant reduction of 5% in the RBC and of 3% in the HCT. There were no differences found between the pre- and post-bariatric concentrations of hemoglobin and ferritin. This may result from adequate supplementation and the patient’s good compliance. Contrary to our results, Vartanoglu et al. found a decrease in ferrum plasma concentration and an increase in ferritin concentration in a group of 100 patients 3 months after LSG or gastric plication. The authors emphasized that ferrum deficiency was common in their country, which might have influenced the results. Additionally, there was no information about the recommended post-bariatric supplementation of ferrum doses [35]. Junior et al. found ferritin levels to be significantly higher in presence of MASLD: 139 µg/L vs 60.9 µg/L [36].

Adequate supplementation of vitamins and nutrients is of utmost importance in attaining optimum results from bariatric surgery and to provide for a good quality of post-bariatric life. It is recommended to monitor the plasma concentrations of ferrum, ferritin, vitamin B12, folic acid, vitamin D3, calcium, among others, in case of deficiency symptoms [22,33]. Potential deficiencies can also be diagnosed by monitoring parathormone levels and bone density [30,31,37].

### Limitations of the Study

The study period included the period of the COVID-19 pandemic, and the number of bariatric procedures performed and the number of patients eligible for the study were limited as a result of IFSO recommendations about postponing bariatric surgery during pandemic. Additionally, the vast majority of the study group included patients after LSG, so the results mainly indicate the changes after LSG.

## 5. Conclusions

Bariatric surgery leads to the amelioration of carbohydrate and lipid metabolism, which includes significant reductions in glucose fasting levels and glycated hemoglobin, triglyceride and low-density lipoprotein concentrations, with a simultaneous increase in high-density lipoprotein concentrations. These changes may be attributed to the reported dietary habit alterations after bariatric surgery, with a significant reduction in red meat consumption and an increase in the consumption of fish and dairy products. To maintain an optimum diet and a changed lifestyle, regular multidisciplinary care that includes bariatric dietitians is recommended.

## Figures and Tables

**Table 1:** Baseline characteristics of the study group.

Data		n	%
Gender	Female	66	75
	Male	22	25
Education	Primary	7	8.0
	Secondary	40	45.5
	Higher	41	46.6
Type of surgery	LRYGB	11	12.5
	LSG	77	87.5

**Table 2:** Body weight, Body Mass Index and selected laboratory parameters before and after the surgery.

Data	Surgery	Min	Max	Median	Q1	Q3
Body weight [kg]	Pre	86.5	260	126	112	145
	Post	61.8	220	87.5	78	106
Body Mass Index [kg/m^2^]	Pre	35.4	82.1	44.3	40.7	48.7
	Post	22.7	59	31.2	28.7	35.7
Glycated hemoglobin [mmol/mol]	Pre	29	62	39	37	41
	Post	25	43	32	31	33
Alanine aminotransferase [U/L]	Pre	8	88	29	20	35.2
	Post	5	49	17	13	21
Aspartate aminotransferase [U/L]	Pre	9	59	22	18	25.2
	Post	10	30	19	15	22
Total protein [g/dL]	Pre	4.9	7.4	5.9	5.6	6.3
	Post	6	8.1	7	6.7	7.2
Total cholesterol [mg/dL]	Pre	127	263	166	147	177
	Post	127	267	169	152	183
High-density lipoproteins [mg/dL]	Pre	29	73	48	43.8	55
	Post	35	121	58.5	50	67.5
Low-density lipoproteins [mg/dL]	Pre	66	193	113	9.5	134
	Post	59	177	104	87	119
Triglycerides [mg/dL]	Pre	51	453	152	121	193
	Post	63	142	108	87	129
Fasting glucose [mmol/L]	Pre	4.3	10.1	5.6	5.0	6.5
	Post	3.9	6.6	4.8	4.4	5.1
Total calcium [mg/dL]	Pre	8.9	90.3	9.6	9.4	9.8
	Post	8.6	10.2	9.8	9.5	9.9
Total ferrum [µg/dL]	Pre	31	193	95	79.8	109
	Post	26	191	110	89	131
Ferritin [ng/mL]	Pre	6	499	74.5	39.8	128
	Post	6	615	79	44.2	124
Folic acid [ng/mL]	Pre	2	19.5	8.4	5.8	12.5
	Post	2.3	32	9.7	7	13.6
Vitamin D3 [ng/mL]	Pre	7.5	66.3	23.4	18.6	26.8
	Post	13.6	68.7	32.4	27	36.4
Vitamin B12 [ng/mL]	Pre	144	1007	452	336	492
	Post	184	1125	459	356	544
Hemoglobin [g/dL]	Pre	10.4	17.3	13.9	13.4	14.9
	Post	10.5	18.5	13.6	12.9	14.7
Red blood cell count [T/L]	Pre	4.3	5.9	4.9	4.6	5.1
	Post	3.6	5.9	4.6	4.3	5
Hematocrit [%]	Pre	34	51	42	40	44
	Post	35	54	41	39	42

## Data Availability

The original contributions presented in the study are included in the article, further inquiries can be directed to the corresponding author.

## List of Abbreviations

WHO	World Health Organization
ERABS	enhanced recovery after bariatric surgery
LSG	laparoscopic sleeve gastrectomy
LRYGB	laparoscopic Roux-en-Y gastric bypass
BMI	Body Mass Index
HbA1c	glycated hemoglobin
FGC	fasting glucose concentration
LDL	low-density lipoprotein
HDL	high-density lipoprotein
TGI	triglyceride
TC	total cholesterol
ALT	alanine aminotransferase
AST	aspartate aminotransferase
TP	total protein
HGB	hemoglobin
RBC	red blood cell count
HCT	hematocrit
TCA	total calcium
TFE	total ferrum
FER	ferritin
FA	folic acid
D3	vitamin D3
B12	vitamin B12
SG	sleeve gastrectomy
RYGB	Roux-en-Y gastric bypass
MASLD	metabolic-dysfunction-associated steatotic liver disease
